# Extracellular vesicles improve embryo cryotolerance by maintaining the tight junction integrity during blastocoel re-expansion

**DOI:** 10.1530/REP-21-0320

**Published:** 2022-02-07

**Authors:** Tabinda Sidrat, Abdul Aziz Khan, Myeong-Don Joo, Lianguang Xu, Marwa El-Sheikh, Jong-Hyuk Ko, Il-Keun Kong

**Affiliations:** 1 Division of Applied Life Science (BK21 Four), Department of Animal Science, Gyeongsang National University, Jinju, Gyeongnam Province, Republic of Korea; 2 Center for Discovery and Innovation, Hackensack University Medical Center, Nutley, New Jersey, USA; 3 Thekingkong Co. Ltd., Gyeongsang National University, Jinju, Gyeongnam Province, Republic of Korea; 4 Institute of Agriculture and Life Science, Gyeongsang National University, Jinju, Gyeongnam Province, Republic of Korea

## Abstract

Cryopreservation is a process in which the intact living cells, tissues, or embryos are preserved at subzero temperatures for preservation. The cryopreservation process highly impacts the survival and quality of the *in vitro*-produced (IVP) embryos. Some studies have highlighted the use of oviduct extracellular vesicles (EVs) to improve the cryotolerance of IVP embryos but the mechanism has not been well studied. The present study unravels the role of *in vitro* cultured bovine oviduct epithelial cells-derived EVs in improving the re-expansion and hatching potential of thawed blastocysts (BLs). The comparison of cryotolerance between synthetic oviduct fluid (SOF) and SOF + EVs-supplemented day-7 cryopreserved BLs revealed that the embryo’s ability to re-expand critically depends on the intact paracellular sealing which facilitates increased fluid accumulation during cavity expansion after shrinkage. Our results demonstrated that BLs cultured in the SOF + EVs group had remarkably higher re-expansion (67.5 ± 4.2%) and hatching rate (84.8 ± 1.4%) compared to the SOF group (53.4 ± 3.4% and 63.9 ± 0.9%, respectively). Interestingly, EVs-supplemented BLs exhibited greater influence on the expression of core genes involved in trophectoderm (TE) maintenance, formation of tight junction (TJ) assembly, H_2_O channel proteins (aquaporins), and Na^+^/K^+^ ATPase alpha 1. The EVs improved the fluid flux and allowed the transport of H_2_O into an actively re-expanded cavity in EVs-cultured cryo-survived BLs relative to control BLs. Our findings explored the function of EVs in restoring the TE integrity, improved the cell junctional contacts and H_2_O movement which helps the blastocoel re-expansion after thawing the cryopreserved BLs.

## Introduction

Cryopreservation has become an integral part of assisted reproductive technology by preserving the superior quality of embryos in a frozen state either by slow freezing or vitrification process (Baust *et al.* 2009). Desirable selection and preservation of supernumerary embryos through the cryopreservation process widely impact the embryo transfer technique (Marsico *et al.* 2019). However, during the conventional process of slow freezing followed by warming and thawing, considerable morpho-functional damage can occur to embryos (Moussa *et al.* 2014). Embryo viability and quality after the freeze-thaw cycle depends on the cellular and molecular competency of the embryos that influence the rate of cryo-survival, pregnancy establishment, and maintenance particularly in case of *in vitro*-produced (IVP) embryos (Moussa *et al.* 2014, Marsico *et al.* 2019).

The *in vitro* culture (IVC) of embryos requires a suitable adjusted condition that can withstand the thermic, osmotic, oxidative, nutritional, and physical stress during cryopreservation (Miller *et al.* 2003). The extent of cryoinjuries varies between *in vivo* and IVP embryos. Usually, IVP embryos are more susceptible to damage due to the culture conditions and have more often shown reduced post-cryo-survival and pregnancy rates than their *in vivo* counterparts (Marsico *et al.* 2019). During *in vivo* production, the oviduct and uterine fluid secretions contain various molecules such as extracellular vesicles (EVs) or exosomes that facilitate the embryo–maternal cross-talks (Bridi *et al.* 2020, Harris *et al.* 2020). These EVs contain the deliverable bioactive molecules such as several mRNAs, DNA, proteins, and lipids which influence the pre-implantation embryonic development and pregnancy maintenance in several ways (Alminana *et al.* 2017, Alminana & Bauersachs 2020). Recently, it was evidenced that EVs derived from *in vitro* cultured bovine oviduct epithelial cells (BOECs)-conditioned media significantly enhanced the *in vitro* BL development and hatching via improving the metabolic flux of the developing embryos (Sidrat *et al.* 2020). Moreover, the co-culture of BOECs or with EVs alone in the IVC media significantly enhanced the cryo-survival rate of bovine embryos (Lopera-Vasquez *et al.* 2017). However, the molecular mechanism through which EVs exert their cryo-protective effects is still unexplored.

The process of freezing and thawing cycle leaves a deleterious effect and particularly causes acute shrinkage and TE damage in the mammalian embryo (Kaidi *et al.* 2001, Camargo *et al.* 2011). The failure of BL re-expansion and cavity collapse are the most common effects observed after cryopreservation (Moussa *et al.* 2014). Embryo quality is usually assessed by the timing and appearance of the blastocoel. The embryos with early cavitation are considered of superior quality, showing proper allocation of the inner cell mass and TE cells, and higher cryo-survival rate (Kaidi *et al.* 2001, Miller *et al.* 2003). The integrity and maintenance of TE epithelium and the membrane permeability barriers are the crucial factors that help the embryo to withstand the extent of cryoinjuries associated with the imbalance of osmotic flux, alteration in the ionic gradient and H_2_O channel proteins as well as thermic, oxidative, and metabolic stress (Kaidi *et al.* 2001, Camargo *et al.* 2011). The maintenance of TE epithelial integrity is dependent on many structural and regulatory components. The transcription factor CDX2 is a critical regulator for the formation of distinct epithelial features of TE (Marikawa & Alarcon 2012). CDX2 expression promotes the epithelial cell adhesion in the TE of expanding blastocyst (BL) by inducing the cadherin-dependent cell–cell adhesion activity (Fleming *et al.* 2001, Strumpf *et al.* 2005). Also to maintain the degree of contact between the neighbouring cells the cellular adhesion molecules such as CD44 exhibited a higher expression during the expansion and hatching of the BL to maintain an extensive cell–cell adhesion around the contact areas of the TE (Lu *et al.* 2002). The expression of all of these genes plays an important role in defining the integrity of TE and in the generation of the cell junctional contacts.

The TE epithelium around the BL is the first-differentiated cell layer formed during the embryogenesis and is primarily engaged to provide a barrier and protection along with facilitating the exchange and transport of small molecules and fluid to generate the blastocoel cavity (Marikawa & Alarcon 2012). One of the critical aspects is the cell differentiation process within TE epithelium is the formation of tight junction complexes at the apicolateral border between the cells (Eckert & Fleming 2008). The tight junction acts as a platform to regulate the intercellular gap junction communication and fluid flux via acting as a paracellular permeability seal between TE cells and the nascent blastocoel cavity (Eckert & Fleming 2008, Marikawa & Alarcon 2012). Luminal pressure inside the fluid-filled cavity of BL is regulated by the actions of tight junction (TJ) multiprotein assembly, which balances the influx and efflux of molecules and accumulation of fluid inside the blastocoel (Chan *et al.* 2019). The components of TJ assembly are encoded by several gene members such as claudin family (*CLDN*), occludin (*OCLN*), junctional adhesion molecule (*JAM*), and cell polarity proteins (Miller* et al.* 2003, Choi* et al.* 2012). *Actinγ2* is the abundant cell polarity protein encoded by the *Actin* gene family, which plays an important role in the molecular organization and coordination of tight and adherens junctional complexes. The coordination of cell junctional complexes is required to seal the cavity that withstands the increase in internal pressure due to fluid influx during blastocoel expansion (Leach *et al.* 2000, Gupta *et al.* 2017, Zenker *et al.* 2018). The transcellular H_2_O movement regulated protein channels aquaporins (AQP) and Na/K-ATPase alpha 1 (ATP1α1) are also involved in blastocoel formation. The proper expression and localization of genes encoding these proteins are important for TJ maturation and function which play an important role during BL development (Miller *et al.* 2003, Camargo *et al.* 2011, Choi *et al.* 2012). Pharmacological blockade or impaired function of these tight junction proteins perturb the TE integrity and lead to reduced cavity size or either collapse of BL (Choi *et al.* 2012, Chan *et al.* 2019). Thus, the intact TJ complex actually drives the BL cavity expansion and maintains the inside luminal pressure via cell stretching (Chan *et al.* 2019).

Based on our previous investigation (Sidrat *et al.* 2020), we speculated that supplementation with EVs could improve the re-expansion and hatching rates of cryopreserved bovine embryos. Here, we aimed to study whether the supplementation of *in vitro* cultured BOECs-derived EVs is effective in withstanding the cryo-damage shocks by maintaining TE epithelialization integrity and influencing the function of the TJ complex during BL re-expansion after thawing. The current study specifically focuses on the effect of EVs supplementation on improving cryo-tolerance by mediating the expression of TJ assembly genes that improves fluid accumulation for blastocoel re-expansion during post-cryo-survival of bovine embryos.

## Materials and methods

All chemicals and reagents used in the assays were purchased from Sigma Aldrich, unless otherwise specified.

### *In vitro* embryo production

Collection of ovaries and *in vitro* production of bovine embryos were performed as described previously (Sidrat *et al.* 2020). Briefly, the ovaries of Korean native Hanwoo cows were obtained from the local slaughterhouse and shipped to the laboratory in physiological saline solution (0.9% NaCl) at 37.5°C. Cumulus oocyte complexes (COCs) with more than three compact layers of cumulus cells were aspirated into TL-HEPES buffered medium supplemented with 100 IU/mL penicillin, and 0.1 mg/mL streptomycin. After successive washing in TL-HEPES medium, COCs were washed and matured in tissue culture medium-199 (TCM-199) supplemented with 10% (v/v) fetal bovine serum (FBS), 1 µg/mL oestradiol -17β, 10 µg/mL follicle-stimulating hormone, 0.6 mM cysteine, and 0.2 mM sodium pyruvate. Following 24 h of *in vitro* maturation, COCs were fertilized* in vitro* with 1 × 10^6^ sperm cells/mL from frozen-thawed semen straws as previously described (Sidrat *et al.* 2020). Following 18–20 h of *in vitro* fertilization (IVF) (IVF-medium supplemented with (tyrode lactate solution supplemented with 6 mg mL^-1^ BSA, 22 mg/mL sodium pyruvate, 100 IU per mL penicillin, and 0.1 mg mL^-1^ streptomycin)), the cumulus cells were removed by repeated pipetting and cleared presumptive zygotes were cultured in a synthetic oviduct fluid (SOF) medium for up to 8 days in a humidified atmosphere of 5% CO_2_ at 38.5°C. For the IVC, SOF medium supplemented with 44 μg/mL sodium pyruvate (C_3_H_3_NaO_3_), 14.6 μg/mL glutamine, 10 IU/mL penicillin, 0.1 mg/mL streptomycin, 3 mg/mL FBS, and 310 μg/mL glutathione, was used for successive development of embryos till to BL stage in four-well plates (Nunc, Roskilde, Denmark) under a humidified atmospheric condition of 5% CO_2_ at 38.5°C.

### Supplementation of extracellular vesicles during* in vitro* culture of embryos

For the supplementation of EVs in the SOF medium, EVs 3% from 100 × diluted DPBS solution (Dulbecco’s phosphate-buffered saline) were added into the SOF media. Briefly, the EVs were isolated and characterized from the *in vitro* cultured BOECs-monolayer derived condition medium. To pellet the EVs, media was centrifuged at 100,000 ***g*** for 60 min at 4°C in a (BECKMAN L8-M; SW41T1 rotor). The pellets were suspended in ice-cold DPBS, aliquoted, and stored at −20 °C. Nano-tracking analysis of 100× dilution of EVs pellet in PBS showed 80–150 nm size with an average concentration of 3 × 10^8^ particles per mL (Sidrat *et al.* 2020). To support the higher embryonic development, the 3% concentration of EVs was supplemented in SOF media based on the previous investigation of the development of bovine embryos (Sidrat *et al.* 2020). The embryo development proceeded to the BL stage in SOF and SOF + EVs supplemented medium cultured until day 7, expanded BLs from SOF and SOF + EVs cultured medium were selected for cryopreservation. Whole embryonic development data were recorded and used for comparative analysis.

### Cryopreservation and thawing procedure

For cryopreservation, an ethylene glycol-based freezing protocol, a conventional gold standard freezing procedure was used. Day 7 expanded BLs (grades 1 and 2; Lindner & Wright 1983) were washed in 0.5% (w/v) BSA solution prepared in DPBS for 5 min. Thereafter incubated for 10 min in a 1.8 M ethylene glycol cryoprotectant medium supplemented with 0.1 M sucrose and 0.5% BSA solution for osmotic equilibration. Afterwards, they were loaded into a 0.25 mL plastic straw. Embryos were slowly frozen using a controlled freezing system (CL-8800i: Cryo-Logic, Blackburn, Victoria, Australia). The embryos were then held at −7°C for 5 min, seeded with a cotton-tipped stick dipped in liquid nitrogen to induce ice crystal formation in cryoprotectant solution either above or below the embryo, then held for another 5 min, and finally cooled down to –35 °C at −0.5°C/min. Subsequently, the straws were plunged into liquid nitrogen. The cryopreserved embryos were carefully transferred to an appropriately labelled goblet immersed in liquid nitrogen and stored until thawing. To thaw the embryos, the straw was held in the air for 10 s, and submerged in a water bath at 37°C for another 20–25 s. Cryo-survived embryo was recovered in SOF and SOF + EVs supplemented medium, after successive washing in SOF media to completely remove the residues of the cryopreservation media. Afterwards, re-expanded or hatched BLs were washed in 1× PBS and used either live or fixed in 4% paraformaldehyde for further experimental analysis. To perform the gene expression analysis, BLs were washed three times in nuclease-free water and stored at –80°C by immediately snap-freezing in liquid nitrogen. Frozen-thawed embryos were cultured in their respective culture medium in which they were cultured before freezing under a humidified atmosphere of 5% CO_2_ at 38.5°C for 24–48 h to obtain the BLs survival and hatching rate.

### Assessment of embryo survival

The survivability of BL was assessed by observing the blastocoel which continued to re-expand after thawing till 24 or 48 h of culturing. The degree of re-expansion was observed after 3 h of post-thawing by analysing the gap between the outer TE of the re-expanding blastocoel and the zona pellucida. Re-expansion and hatching rates were recorded at 24–48 h of culture. The percentage of BL re-expansion and hatching was observed over the total number of survived BL from SOF and SOF + EVs treated group.

### RNA isolation, cDNA synthesis, and PCR

The total RNA was extracted by using the PicoPure RNA extraction kit (ThermoFisher) according to the manufacturer’s instruction. cDNA was synthesized from the extracted RNA using iScript reverse transcriptase (BioRad). Relative quantification for real-time quantitative PCR (qRT-PCR) analysis was conducted as described previously (Sidrat*et al.* 2019). Briefly, for qRT-PCR, SYBR Green master mix using the Cycler BioRad system was used for the analysis of relative mRNA abundance of all genes. Normalization of threshold (Ct) values of all tested genes was done with (Ct) values of GAPDH. The conditions for PCR amplification were as follows: template cDNA denatured at 94°C for 5 min, followed by 40 cycles of 94°C for 30 s, each gene-specific primer annealing temperature for 30 s, elongation at 72°C for 30 s. Negative controls were also run with each group of samples comprising the PCR reaction mixture without the addition of the cDNA template. For the analysis of mRNA expression pattern, the reaction was performed in triplicates with *n*  = 5 BLs per group from three individual sets of independent experiments. The primers used for RT-PCR and qRT-PCR are listed in Supplementary Table 1 (see section on supplementary materials given at the end of this article).

### ROS assay

The production of reactive oxygen species (ROS) analysis was performed in cryo-surviving BLs cultured in SOF and SOF + EVs supplemented groups. To perform the ROS assay, the fluorescent probe 2’,7’-dichlorodihydroﬂuorescein diacetate (DCHDFA) product from Sigma Aldrich (D-6883) was used. The assay was performed by making the stock solution of DCHDFA in DMSO. The stock solution was further diluted in PBS/PVP solution to obtain the working concentration. The cryo-survived BLs from SOF and SOF + EVs cultured groups were incubated in 10 mM/mL concentration of DCHDFA solution at 37°C for 30 min. After that, BLs were washed thoroughly in PBS/PVP solution and visualized under a confocal laser microscope (Olympus) for the detection of ROS levels.

### TUNEL assay

The effect of freezing and thawing on cellular DNA fragmentation was determined by using the In Situ Cell Death Detection Kit (Roche Diagnostics Corp.). A terminal deoxynucleotidyl transferase 20-deoxyuridine, 50-triphosphate nick-end labelling) assay was performed using channel absorbance at 536 nm and emission at 623 nm. For the detection of TUNEL-labelled positive nuclei in 24 h post-thawed re-expanded BLs cultured in SOF and SOF + EVs groups; BLs were first washed with PBS/polyvinylpyrrolidone (PVP) solution and fixed in 4% paraformaldehyde (PFA) for 15 min. After fixing, the BLs were washed and performed permeabilization (0.5% (v/v) Triton X-100 and 0.1% (w/v) sodium citrate) for 30 min at room temperature (RT). Following permeabilization, BLs were incubated with TUNEL assay kit cocktail for 1 h at 37°C under preventive light conditions. To stop the reaction, BLs were washed with PBS/PVP solution and thereafter counterstained with DAPI (5 min) for detection of nuclei in each cell. Washed BLs were mounted on a glass slide and the images were recorded by epifluorescence microscope (Olympus). TUNEL-labelled positive nuclei from individual BL were analysed by using Image J software (NIH). The average percentage was obtained by dividing the total cell number (stained with DAPI).

### Immunoflorescence staining

For the immunofluorescence staining, post-thawed re-expanded or hatched BLs from SOF and SOF + EVs cultured groups were collected and fixed with 4% (w/v) PFA solution. BLs were washed three times with PBS/PVP solution and permeabilized with 0.5% Triton X-100. After washing, BLs were left in blocking solution (3% BSA in PBS/PVP) for at least 2 h at RT. Following blocking, incubated with primary antibodies (CDX2, CD44, AQP3, and ATP1α1, diluted in PBS/PVP solution) at 4°C for overnight. After successive washing, incubated with secondary antibody either conjugated with FITC or TRITC (diluted in PBS/PVP solution) at RT for 1 h. To remove the background staining and left over-residual stains, BLs were extensively washed with PBS/PVP solution and incubated with DAPI for 5 min at RT for counterstaining of nuclei. Washed BLs were then mounted on a glass slide with a drop of glycerol and overlaid by a coverslip. For the expression analysis, confocal imaging was performed by using a confocal laser microscope (Olympus). The antibodies used for immunofluorescence are listed in Supplementary Table 2.

### Tight Junction permeability assay

The group of post-thawed 24 h re-expanded BLs from SOF and SOF + EVs groups were collected and incubated with 1 mg/mL 4 kDa FITC-dextran (Sigma-Aldrich) in their respective culture medium at RT for 10 min under protected light condition. Thereafter, BLs were immediately washed and tight junction permeability was then visualized by the presence of fluorescence intensity within the blastocoel under an epifluorescence microscope (Olympus). The presence of high and low fluorescence signal intensities was then quantified using Image J software.

### Statistical analysis

The effect and comparison of culture medium on embryo development, post-thaw survival, and hatching were analysed by using SPSS software version 18.0 (IBM Corp.). All embryonic percentile data from triplicate sets of four separate experiments are represented as the mean ±s.e.m. Triplicate sets of experiments were carried out to analyse the imaging data and for representative image single BL image was shown from the individual group. The mean fluorescence intensities from all the imaging data were quantified per BL (*n*  = 15–20) from each group and Image J software (National Institute of Health, Bethesda, MD, USA) were used to measure all the histogram values. The all represented graphical data shows the mean ± s.e.m. values obtained from the triplicate series of experiments. For the comparative analysis of various genes expression in each group, a Student’s *t-*testwas used. All the imaging data and genes expression statistical analysis were performed using GraphPad Prism 6.0 software package. *P* values designated as: *P< 0.05; **P< 0.01; ***P< 0.001 were considered significant differences.

### Experimental design

#### Experiment 1

**Figure 1:**
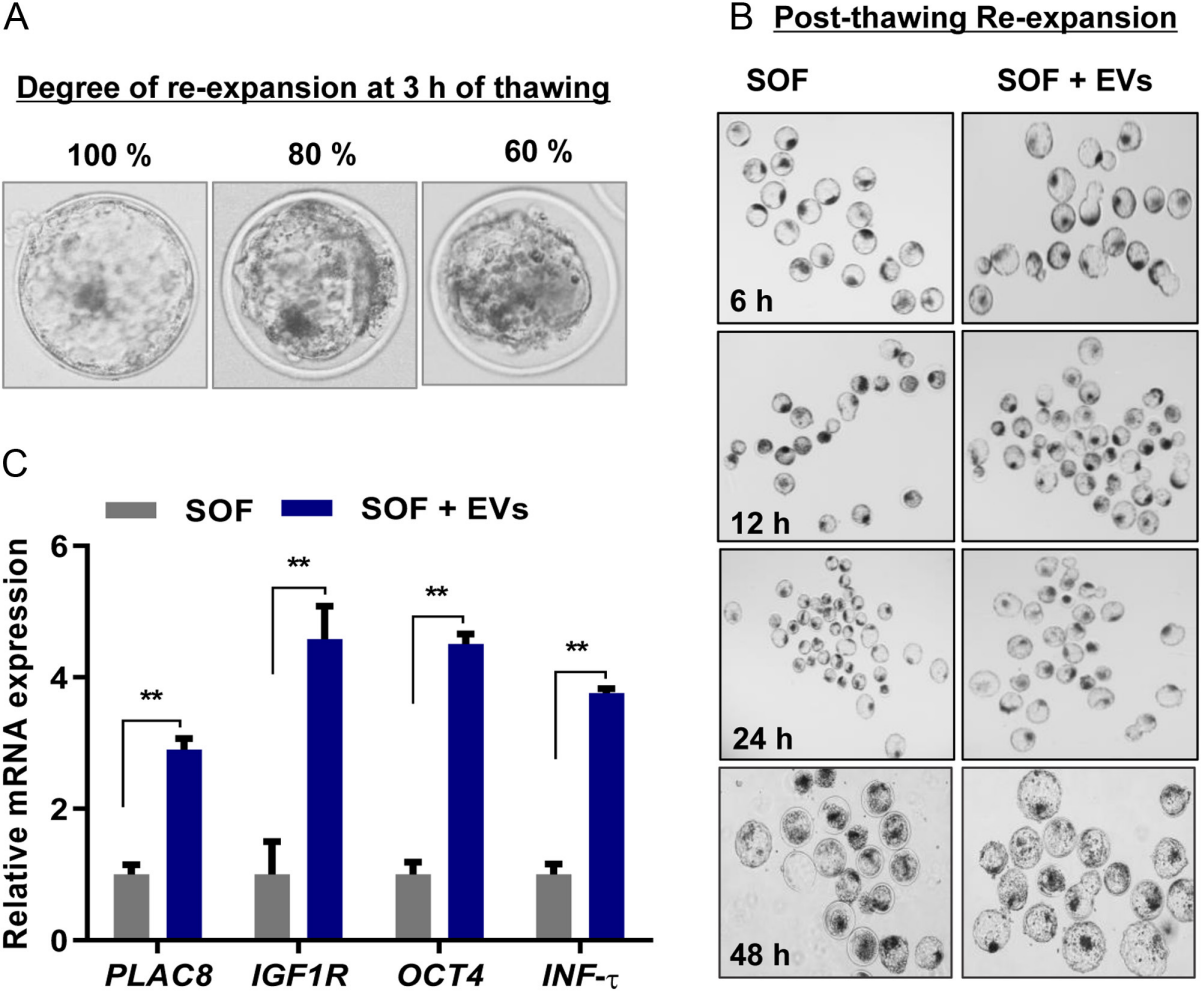
Re-expansion, hatching, and expression of developmental competence genes in post-thawing bovine BLs cultured in SOF with or without EVs. (A and B) Representative bar graph shows the survival and hatching percentage of BLs cultured in SOF and SOF + EVs medium. Post-thawing data are presented as a result of means ± s.e.m. of 16 replicates with (*n*  = 5 to 8 BLs/per group) cryopreserved each time. (C) BLs images after 3 h of thawing showing the degree of re-expansion 100% (seen no space between the TE and ZP; 80% (seen narrow space between TE and ZP); 60% (wider gap between TE and ZP). (D) Images of BLs undergoing re-expansion at 0, 12, 24, and 48 h in their indicated culture medium. (E) Transcript abundance for developmental competence-related genes expression in SOF and SOF + EVs supplemented cryo-survived BLs. For qRT-PCR analysis *n*  = 5 BLs/group used in triplicate experiments. ** *P*  < 0.01; **** *P*  < 0.0001 denotes significant difference.

To determine the effect of EVs addition during the freeze‒thaw cycle, embryos were cultured in the presence and absence of EVs supplemented media and on day 7 high-quality BLs were selected from each group for cryopreservation. BL re-expansion is a predictive hallmark for the assessment of better cryo-tolerance of frozen‒thawed embryos. Therefore, the degree of blastocoel re-expansion was observed 3 h post-thawing, and the morphology of BLs re-expansion rate was recorded after 6, 12, 24, and 48 h in control without EVs (SOF) and SOF + EVs groups. The BLs survivability was assessed by observing the blastocoel during re-expansion after thawing and the hatching rate was recorded at 24 and 48 h of post-thawing in SOF and SOF + EVs group. Furthermore, the effect of EVs supplementation on improving the quality of cryo-survived embryos was analysed in terms of expression of development competence-related genes (Fig. 1).

#### Experiment 2

**Figure 2:**
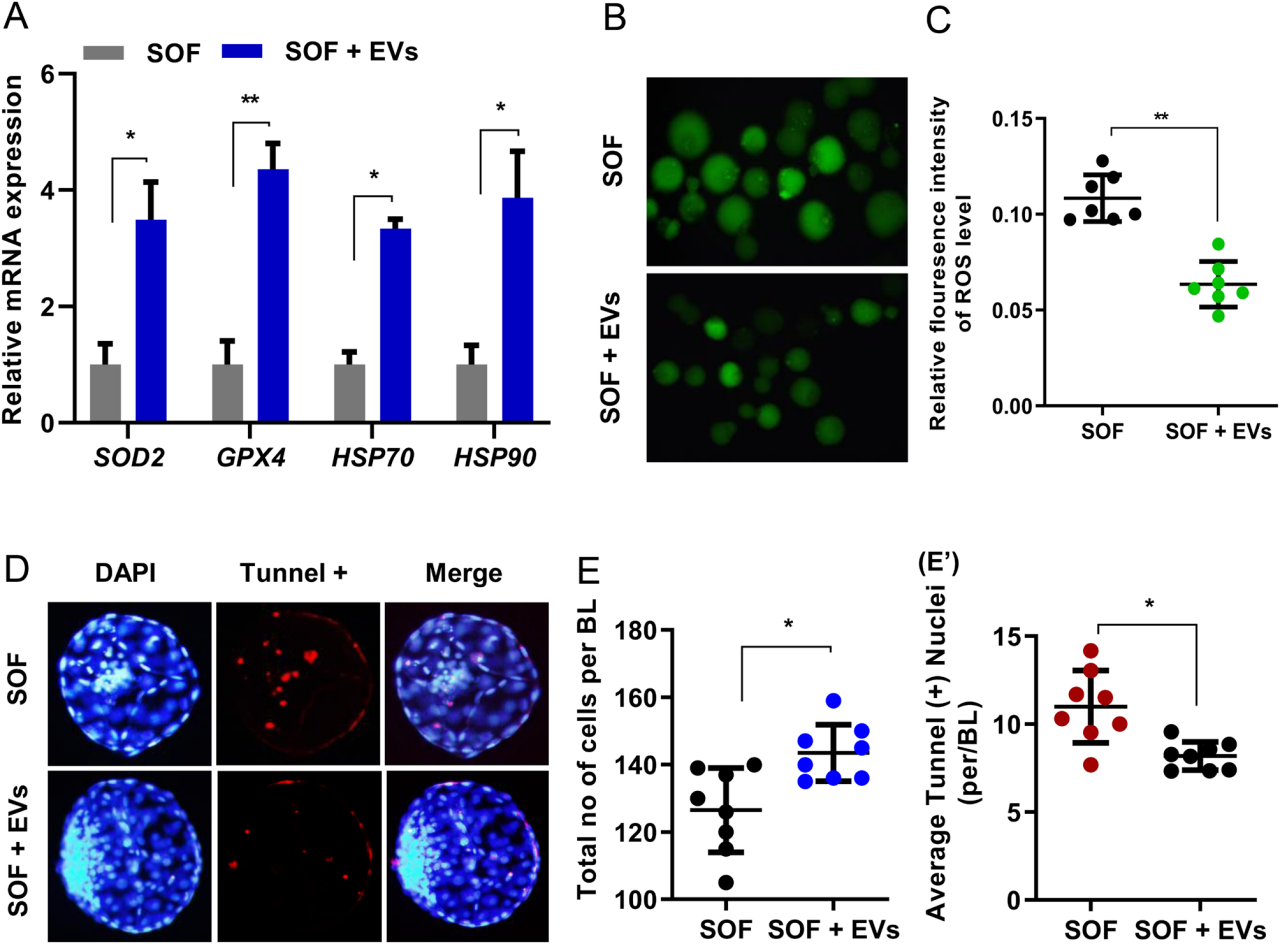
Response to cellular stress in post-thawing bovine BLs cultured in SOF without or with EVs. (A) Relative mRNA level of cell stress genes (MnSOD2, GPX4, HSP70, and HSP90) in SOF and SOF + EVs supplemented cryo-survived BLs. (B and C) ROS assay (C) Fluorescence images showing the TUNEL-positive cells (red) and DAPI (blue) in cryo-survived BLs from SOF and SOF + EVs supplemented groups. White arrows indicated the apoptotic cells in nuclei. (D, E, and E′). Average total cell nuclei and TUNEL (+) nuclei per BLs in SOF and SOF + EVs groups. Data indicated means ± s.e.m. from three independent experiments with (*n*  = 15 BLs) per group in each set of TUNEL assays. For mRNA expression analysis, *n*  = 5 BLs/group was used in triplicate experiments. * *P*  < 0.05; ** *P*  < 0.01 denotes significant difference. Original magnification of images was 100×.

To evaluate the impact of EVs supplementation on the quality of cryo-surviving embryos and their response to cellular stress, the changes in mRNA levels of antioxidant enzymes such as superoxide dismutase (SOD2), glutathione peroxidase (GPX4), and heat shock proteins (HSP70 and HSP90) in BLs cultured in SOF and SOF + EVs groups were analysed. The response of cryo-survived embryos to the cellular stress was further examined by ROS and apoptotic assay (Fig. 2).

#### Experiment 3

**Figure 3:**
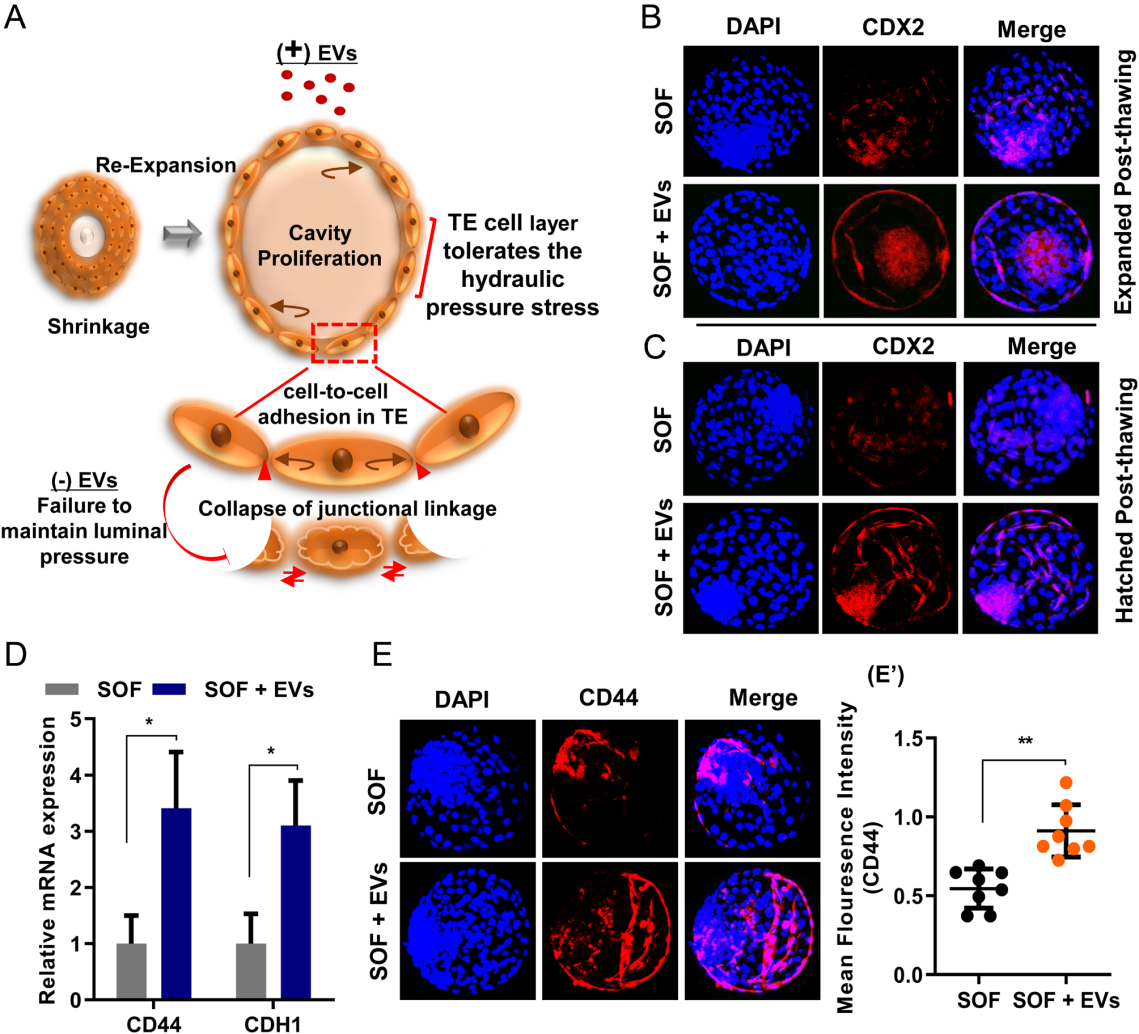
Expression of TE and cell junction proteins in post-thawing bovine BLs cultured in SOF without or with EVs. (A) Proposed physical model showing the EVs supplementation effect during blastocoel re-expansion. (B and C) Immunofluorescence imaging showing the expression of CDX2 in re-expanded and hatched BLs at 24 h of post thawing in SOF and SOF + EVs group. (D) Relative mRNA abundance (mean ± s.e.m.) of genes involved in cell junction establishment in post-thawed BLs from SOF and SOF + EVs group (*n*  = 5 BLs per group from three independent experiments). (E) Confocal images show the CD44 expression in BLs at 24 h of post-thawing in their representative cultured groups. (E′) Bar graph shows the quantification of signal intensities of CD44 expression. Imaging data are presented as the result of mean ± s.e.m. from triplicate experiments (*n*  = 15 BLs per group). *P  < 0.05; **P  < 0.01 indicates significant difference.

The morphological feature of BLs with enlarged cavity size during expansion is predominantly attributed to the well-maintained and stabilized TE epithelial lining, which plays an important role in shaping the expanded BL. Thus the aim of this experiment was to assess the effect of EVs on the integrity of TE epithelium and cell-junctional contact between the TE epithelial cells. We analysed the TE marker CDX2 expression in the expanded and hatched cryo-survived BLs. To check the stabilization of cell-to-cell contact, the mRNA and protein expression of cell adherens junction marker *CD44* and cadherin (*CDH1*) were analysed (Fig. 3).

#### Experiment 4

**Figure 4:**
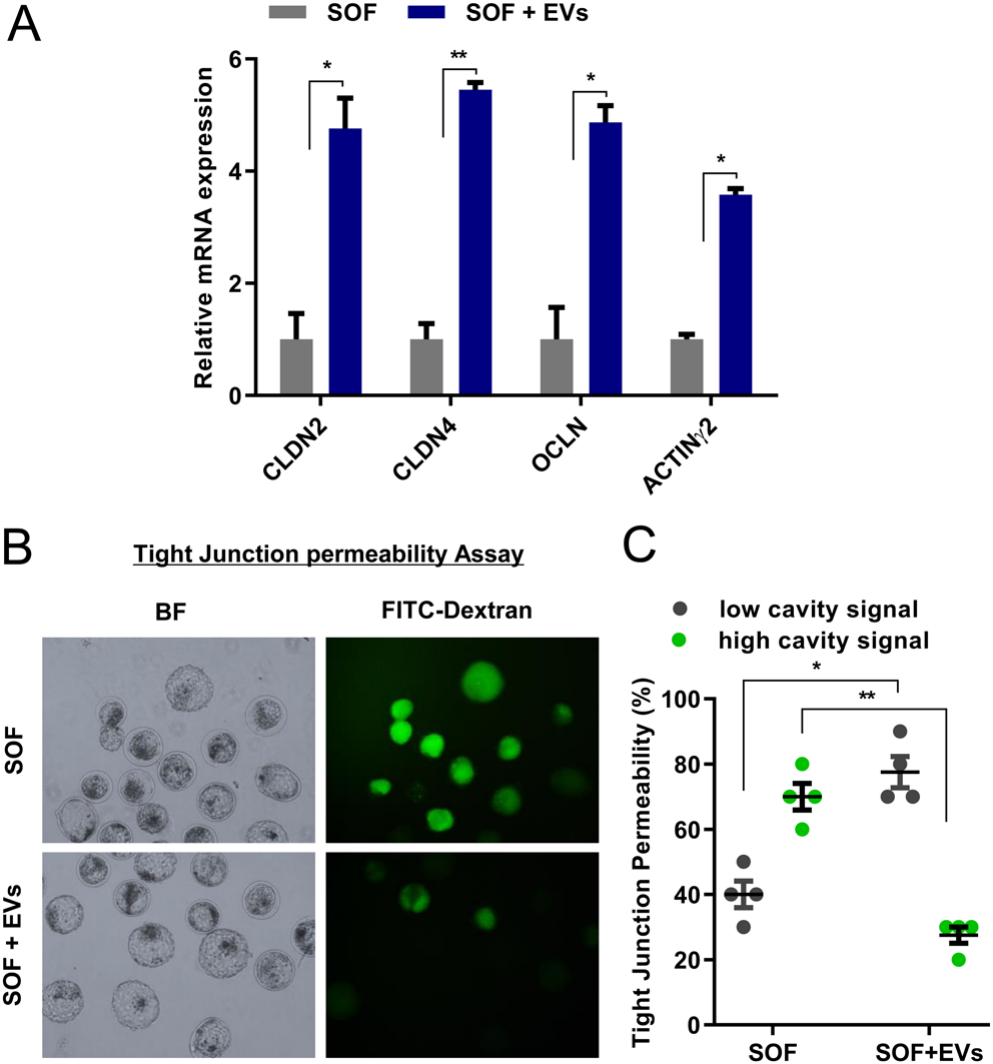
Expression of cell junctional genes and TJ sealing assay in post-thawing BLs cultured in SOF without or with EVs. (A) Relative transcript abundance for cell junctional genes (*CLDN2, CLDN4, OCLN,*and* ACTINγ2*) in SOF and SOF + EVs groups. Bar graphs presented the data of means ± s.e.m. from three separate sets of experiments including (*n*  = 5) BLs per group in each replicate. *P  < 0.05; **P  < 0.01 denotes significant difference among the groups. (B) Representative images of BLs cultured in SOF and SOF + EVs and submitted to 4 kDa FITC-labelled dextran assay for 24 h to assess TJ sealing. (C) Proportion of low and high cavity fluorescent signal in BLs cultured in SOF and SOF + EVs and then treated with 4KDa FITC labelled dextran for 24 h. Data presented in bar graphs as means ± s.e.m. from triplicate experiments in each group (*n*  = 15). Original magnification was 100×.

The aim of this experiment was to analyse the effect of EVs on the expression of TJ genes and the assessment of TJ permeability. We speculated that the function or integrity of these TJ complexes would be compromised during freeze and thaw cycles of the cryopreservation process. Therefore we evaluated the mRNA expression of genes involved in TJ assembly, that is, *claudin* family (*CLDN2, CLDN4*), *occludin* (*OCLN*), *Actinγ2,* and TJ permeability by FITC-dextran assay in cryo-survived BLs (Fig. 4).

#### Experiment 5

**Figure 5:**
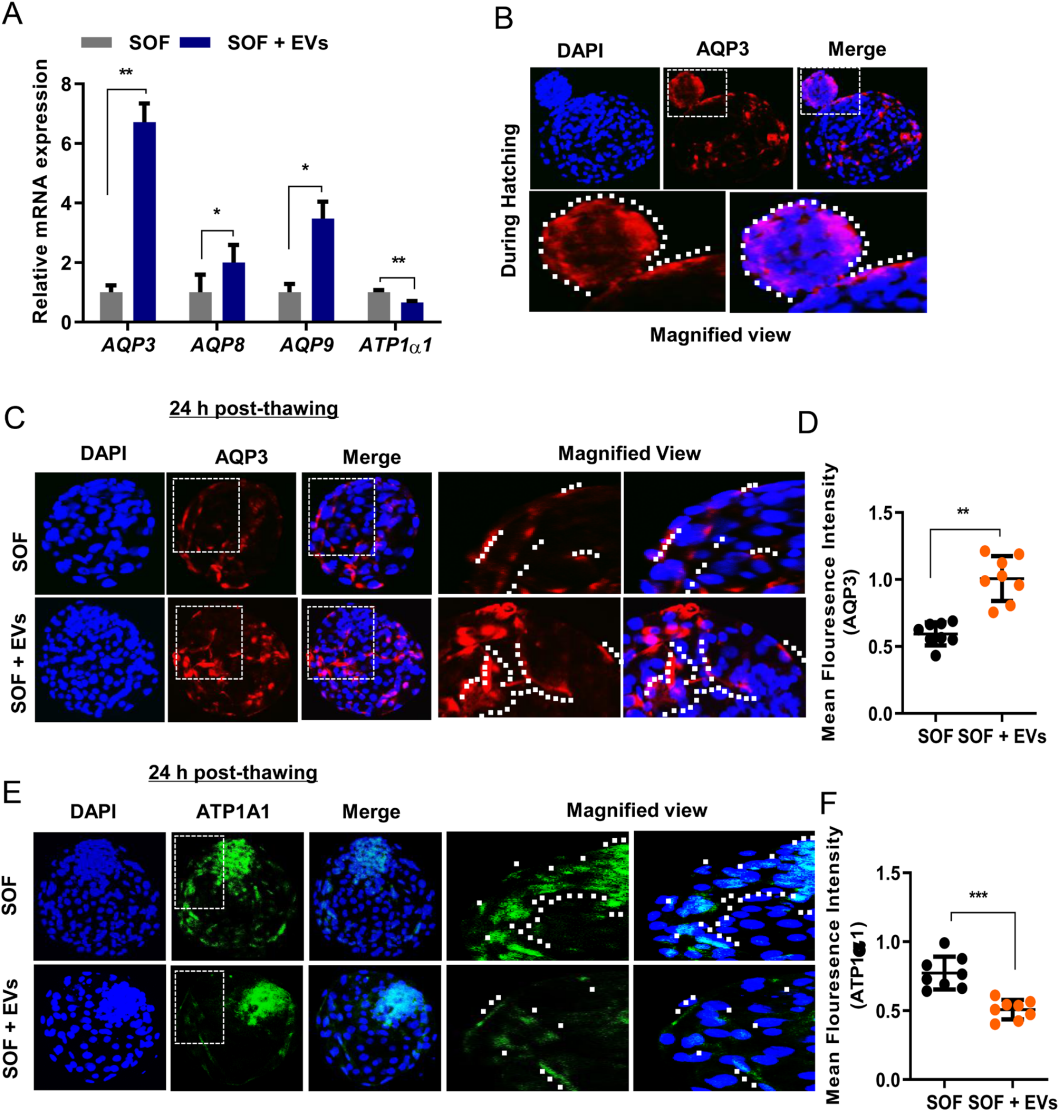
Expression of AQPs and ATP1α1 in post-thawing bovine BLs cultured in SOF without or with EVs. (A) Relative mRNA analysis shows the expression of *AQPs* (*AQP3, AQP8, and AQP9*) and Na^+^, K^+^ ATPase subunit alpha 1 (*ATP1α1*) genes at 24 h post-thawed BLs from SOF and SOF + EVs cultured groups. (B) Imaging analysis shows the protein expression of AQP3 during hatching. (C and D) Confocal imaging shows the expression of AQP3 in re-expanded post-thawed BLs cultured in the indicated medium. Bar graph shows the quantification of fluorescence signal intensities from each representative group. (E and F) Immunofluorescence of ATP1α1 in cryo-survived BLs cultured in SOF and SOF + EVs supplemented medium. Fluorescence signal intensities are quantified in the bar graph. Imaging data presented as mean ± s.e.m. of three replicates. *P  < 0.05; **P  < 0.01; ***P  < 0.001; indicates significant difference. Image magnification 100×.

In this experiment we analysed the effect of EVs on the expression of water and solute transport genes and proteins. We determined the relative mRNA transcript of H_2_O channel regulated AQPs and Na/K-ATP1α1 genes and protein expression of AQP3 and ATP1α1 in SOF and SOF + EVs cultured cryo-survived BLs (Fig. 5).

## Results

### Effect of EVs supplementation on post-thaw survival, hatching, and developmental competence-related genes in re-expanded bovine blastocyst

The presence of EVs in culture medium (SOF + EVs) significantly improved the BLs development (42.0%) and expansion rate (61.4%) compared to the control group (28.2 and 41.8%, respectively) cultured in SOF without EVs (SOF) (Table 1). Later the effect of EVs on BLs re-expansion was observed after 3 h of post-thawing. Interestingly, the SOF + EVs group, BLs showed a rapid re-expansion recovery rate after cryo-shrinkage (Fig. 1A). It was observed that SOF + EVs supplemented group re-expanded faster than the SOF without EVs addition group. The EVs supplemented BLs mostly achieved 80–100% of cavity re-expansion after 3 h of thawing compared to the SOF group (Table 2). The images recorded after 6, 12, 24, and 48 h of post-thaw showed that the BLs recovered after cryo-shrinkage and the SOF + EVs supplemented group re-expanded better and faster than the SOF without EVs added group (Fig. 1B). There was no difference in the BLs survivability between the SOF and SOF + EVs (87 ± 0.7; 89.1 ± 0.8) groups respectively. While SOF + EVs group showed a promising difference in their ability of hatching within 24 h of thawing relative to the SOF group which mostly hatched at 48 h of thawing (Table 3). Furthermore, the cryo-survived BLs cultured in the presence of EVs supplemented media exhibited significantly higher (*P*  < 0.01) mRNA transcript level of development competency markers *PLAC8, IGF1R, OCT4,* and *INFτ* relative to the non-EVs supplemented control counterpart BLs (Fig. 1C). These observations suggest that the addition of EVs significantly improves the cavity re-expansion and hatching rate and also promotes an increase in the selected developmental competence-related genes in cryo-survived bovine embryos produced *in vitro*.

**Table 1:** Effect of EVs supplementation on bovine embryonic development during in vitro.

	Groups	
	SOF	SOF + EVs
Oocytes, *n*	649	608
Speculated zygote, *n*	600	565
Cleaved embryos, *n* (%)	470 (78.3 ± 0.61)	442 (78.06 ± 0.96)
Total BLs, *n* (%)	169 (28.2 ± 0.77)^a^	236 (42.0 ± 1.36)^b^
Expanded BLs, *n* (%)	71 (41.8 ± 5.28)^a^	142 (61.4 ± 2.03)^b^

^a,b^*P* > 0.05 with different superscripts indicates the significant difference.

**Table 2:** Degree of re-expansion after 3 h of thawing in SOF and SOF + EVs cultured BLs.

	SOF, % (*n*)	SOF + EVs, % (*n*)
Degree of re-expansion (%)		
60	31.8 ± 3.6 (28)	28.0 ± 2.4 (32)
80	15.9 ± 1.5 (14)	20.1 ± 1.2 (23)
100	5.6 ± 0.9 (5)^a^	19.2 ± 2.2 (22)^b^
Total re-expanded BLs	53.4 ± 3.4 (47)^a^	67.5 ± 4.2 (77)^b^
Total survived BLs (*n*)	88	114

^a,b^*P* > 0.05 with different superscripts indicates the significant difference.

**Table 3:** Post-cryopreservation survival and hatching rate of thawed BLs cultured in the presence and absence of EVs supplementation after 24 and 48 h.

Groups	Thawed BLs (*n*)	Hatching rate (%)		Total hatching rate (%)
		24 h	48 h	
SOF	101	27 (29.8 ± 2.2)^a^	29 (33.9 ± 3.0)^a^	56 (63.9 ± 0.9)^a^
SOF + EVs	128	75 (65.4 ± 2.8)^b^	22 (19.4 ± 2.6)^b^	97 (84.8 ± 1.4)^b^

^a,b^ *P*  > 0.05 with different superscripts indicates the significant difference.

### EVs supplementation improves tolerance against cellular stress in the cryo-survived embryos

Low embryo quality after cryopreservation is probably due to the cellular stress occurring during the procedure. The qRT-PCR analysis indicated that mRNA transcript of antioxidant enzymes SOD2, GPX4, and heat shock proteins, HSP70 and HSP90 was significantly higher in the BLs cultured in the presence of EVs supplemented media than the BLs cultured without the addition of EVs. The increased relative mRNA level of these genes suggests that the supplementation of EVs in the culture media might help to reduce the effect of cellular stress in BLs undergoing freeze-thaw cycle relative to their control counterpart (Fig. 2A). This result was further confirmed by the analysis of accumulated ROS, which showed low fluorescent signals in the EVs additive group relative to the control group (Fig. 2B and  C). This observation suggests that the addition of EVs to the culture media increases the expression of antioxidant genes and effectively counteracts the accumulated ROS levels in the cryopreserved BLs. The elevated HSPs in turn helps to reduce the DNA damage response caused by physiological stress during the whole process of cryopreservation which was confirmed by apoptotic assay. This result shows a higher cell nuclei count and significantly low (*P*  < 0.05) apoptotic ratio in the SOF + EVs group as indicated by the percentage of TUNEL-labelled positive nuclei per BL. In contrast, the SOF control group showed a comparatively low cell nuclei count and higher apoptotic index (Fig. 2D, E, and E′). Taken together, these results suggest that EVs addition in the culture media improves cryo-tolerance by inducing the expression of antioxidants and HSPs to enhance the developmental potential of cryopreserved BLs after thawing.

### Effect of EVs supplementation on the maintenance of TE integrity in post-thawed embryos

We hypothesized that the supplementation of EVs in the culture media maintained the TE integrity during re-expansion of the blastocoel cavity by stabilizing the cell-to-cell junctional contacts in the TE epithelial cells. The stability in the cell-to-cell junctional contacts may sustain the mechanical and physical stress shocks during the cryopreservation process (Fig. 3A). CDX2 is an important early TE marker. Therefore, the localization of CDX2 protein and continuity of its expression along the TE epithelium weres assessed to examine whether the feature of TE epithelial lining was restored in cryo-survived BLs after thawing. The immunofluorescence analysis showed that CDX2 in EVs supplemented group was uniformly expressed around the whole TE cell lining in re-expanded and hatched BLs. In contrast, the SOF cryo-surviving BLs displayed a reduced and intermittent expression pattern (Fig. 3B and  C). These results suggest that EVs supplementation in the culture media stabilizes the TE epithelial lining in the re-expanded blastocoel cavity by inducing a continuous expression of CDX2 protein compared to the control group. During subsequent freeze and thawing, the cells in the TE epithelium might lose their junctional connections and may not firmly adhere to each other to sustain the TE integrity. Thus we examined the cell adherens junction markers *CD44* and cadherin (*CDH1*) expression and found higher mRNA levels (*P*  < 0.05) of *CD44* and *CDH1* in BLs cultured in the presence of EVs relative to the control group (Fig. 3D). Furthermore, imaging analysis revealed that CD44 is expressed as a continuous belt around the TE epithelial cells and was more enriched at the cell-to-cell contact in the re-expanded BLs cultured in the EVs supplemented media relative to the control BLs (Fig. 3E and E′). These observations suggest that EVs supplementation strengthens junctional connections after thawing by influencing the adherens junction genes expression and maintains a seamless epithelial lining around the expanded blastocoel cavity.

### EVs addition stabilizes the tight junction complexes in cryo-survived embryos

The formation of paracellular sealing via functional TJ complexes is another important morphological feature of the TE epithelial lining, which allows the development of expanded BL cavities. The qRT-PCR analysis revealed a higher mRNA abundance of TJ genes (*CLDN2*, *CLDN4*, *OCLN*, and *Actinγ2*) in the EVs supplemented group compared to the BLs cultured without EVs addition (Fig. 4A). This suggests that reduced expression of TJ genes leads to perturbed paracellular sealing and might be responsible for the delayed expansion of the BLs. Further, we analysed the permeability barrier of TJ by using a 4 kDa FITC-dextran assay. An exceptionally low percentage of EVs supplemented BLs (22%) was permeable to FITC-dextran compared to control BLs (60%) (Fig. 4B and  C). This shows that the function of TJ was impaired in cryo-survived BLs, whereas the supplementation of EVs maintained the integrity of TJ complexes during the freeze‒thaw cycle by upregulating the expression of TJ assembly genes and leading to fully expanded BLs.

### Supplementation of EVs influences the fluid influx during re-expansion of cryo-thawed embryos

In addition to the establishment of functional TJs, the maintenance of fluid flux such as H_2_O movement is crucial for the accumulation of fluid within the expanded BL cavity. Interestingly, the qRT-PCR analysis revealed that expression of *AQPs* was significantly upregulated (*P*  < 0.05; *P*  < 0.01) whereas the *ATP1α1* was downregulated (*P*  < 0.01) in the SOF + EVs supplemented group compared to the SOF group (Fig. 5A). We also examined the water channel protein AQP3 expression during hatching of the non-cryopreserved BLs which was intensely distributed in the TE epithelial lining of the emerging BL (Fig. 5B). This suggests that fluid accumulation generates a threshold force for the re-expansion and hatching of the BLs. Furthermore, imaging analysis revealed that AQP3 signals were strongly distributed along the TE epithelial lining of the EVs supplemented BLs relative to the control cryo-survived BLs (Fig. 5C and  D). Similar to the qRT-PCR result, the immunofluorescence analysis of ATP1α1 further confirmed that its expression was reduced in SOF + EVs supplemented group compared to the SOF group (Fig. 5E). Quantification of fluorescence signal intensities was measured and are presented in (Fig. 5F). These observations suggest that EVs supplementation influences the fluid flux and allowes the inward fluid movement during BLs re-expansion and hatching.

## Discussion

The ability of the embryo to undergo shrinkage and re-expansion during cryopreservation revealed the positive influence of EVs addition in the cultured medium which improved cryo-tolerance in freeze‒thawed embryos. Our results of bovine embryo cryopreservation demonstrated that (i) EVs supplementation led to a higher survival rate, active re-expansion and hatching of the BLs, and reduced the effect of cellular stress by inducing the expression of antioxidant marker genes; (ii) EVs supplementation effectively restored the TE epithelial integrity during the freeze‒thaw cycle; (iii) EVs addition improves the function of TJ complexes by up-regulating the expression of tight junction assembly genes; (iv) EVs supplementation improves the transcellular movement of H_2_O and solutes across the TE epithelium by influencing the expression of genes crucial for fluid accumulation.

The presence of EVs in the culture media had a significant effect on improving the development and quality of the IVP embryos (Lopera-Vasquez *et al.* 2016, 2017, Sidrat *et al.* 2020). Here, the addition of EVs in the culture media not only improved the total BLs development rate but also the EVs supplemented cryo-survived embryos showing a faster BLs re-expansion recovery rate. These observations reflect that the quality of embryos and their capacity of cryo-tolerance was improved. The total BLs hatching rate during the 48 h culture period after thawing was significantly higher in the SOF + EVs supplemented group than the SOF group. The lower embryo development rate as well as the reduced post-thaw hatching ratio in SOF alone group relative to the SOF + EVs group might reflect the deficiencies of the IVC system that eventually affected the quality of BLs as evidenced by Lonergan (2007). Few studies have documented that the co-culture of EVs derived from the BOECs is highly effective in compensating the deficiencies of the IVC media and enhancing the quality of IVP embryos (Lopera-Vasquez* et al.* 2017, Sidrat *et al*. 2020). The impact of culture conditions on several developmental potential related genes expression that influences the quality of embryos was already reported (Rizos *et al.* 2008, Sadeesh *et al.* 2016). The positive effect of EVs on enhancing the relative transcript abundance of important developmental competence-related genes *PLAC8, IGF1R, OCT4,* and *INFτ* whose functions enhance the developmental potential provides the possible molecular explanation that how EVs supplementation improves the quality of cryo-survived embryos.

The process of cryopreservation hugely impacts the quality of the survived embryos in terms of cryo-tolerance towards the metabolic, oxidative, and physical stress during the freeze-thaw cycle and affects the later *in vitro* development (Kaidi *et al.* 2001). These cryo-damage effects were notably observed in the case of SOF alone group whereas the SOF + EVs group significantly respond to these changes by potentially increasing the transcripts level of antioxidants and HSPs in the cryo-survived BLs. The increased level of antioxidants *SOD2* and *GPX4* in SOF + EVs group helps to neutralize the free oxygen radical species produced as a result of an imbalance in the oxidation–reduction process that occurred during the freeze‒-thaw cycle. HSPs are often known as heat shock stress proteins and their expression is increased as a means to enhance the post-thaw cell viability that is induced in response to unfavourable conditions (Hansen 2007, Zhang *et al.* 2011). The elevated expression of HSPs in EVs supplemented group recapitulated the previous findings that HSPs are the most abundant proteins present in the bovine oviduct EVs (Alminana *et al.* 2017) that may transfer these proteins to the embryos. The presence of higher transcript abundance of HSPs might reverse the DNA damage response that occurred during the stressful process of cryopreservation and thus lower the cellular apoptotic index relative to the SOF alone group. The observations from the first two experiments highlighted that *in vitro* embryos were able to uptake the EVs from *in vitro* cultured media that benefits the embryo BL rate, higher cryo-survival potential and quality, and further potentiate the role of EVs in mediating the cross-talk between the embryo and its environment (Bridi *et al.* 2020).

TE surrounds the actively expanding blastocoel, therefore maintenance of its functional integrity is a crucial factor for embryo survival (Choi *et al.* 2012). Proper development of TE epithelium is important for cavity formation and depends on the adequate establishment of TJ sealing, balance osmotic gradient, and water flux into the cavity (Marikawa & Alarcon 2012). Alteration in any of these TE functions would result in failure of the BL cavity formation (Miller *et al.* 2003, Choi *et al.* 2012). It is well established that early TE differentiation marker CDX2 is indispensable for the proper functioning and maintenance of TE (Wu *et al.* 2010). Based on our observation, after thawing the average proportion of embryos cultured in the SOF alone group showed a remarkably reduced expression pattern of TE epithelial marker. The EVs supplemented group effectively restored the functional integrity of TE epithelial lining by promoting the expression of TE differentiation marker CDX2. This result is supported by the previous observation that EVs secreted by the donor oviductal cells improved the cellular differentiation in the TE layer and accelerates the birth rate after an embryo transfer in mice (Qu *et al.* 2017). Previously, it was demonstrated in the mouse embryonic study that CDX2 is required for the cell adhesion and junctional sealing that maintain the TE epithelial integrity around the expanding blastocoel (Strumpf *et al.* 2005, Wu *et al.* 2010). In this study the major difference we observed after thawing was that the EVs supplemented group had robustly expanded BLs with enlarged cavity than the BLs cultured without the EVs addition. Thus it might be possible that the reduced expression of CDX2 is responsible for reduced cell-cell contact and insufficient function of junctional components in SOF freeze-thawed BLs while the addition of EVs restore their function by elevating the expression of CDX2.

Moreover, the cell-to-cell adhesion in TE epithelium is greatly affected during the freezing procedure that plays an important role in TE stability (Kirschner *et al.* 2011). The decreased expression of cell adhesion marker CDH1 and CD44 in cryo-survived BLs of the SOF group explains that cell-to-cell contact is reduced. Thus the TE of the re-expanded BLs cannot sustain the pressure of accumulated fluid during re-hydration and results in failure of BL cavity expansion. While the supplementation of EVs maintained the integrity of TE epithelium by up-regulating the expression of cell adhesion markers that kept the cell-cell contact intact in the majority of BLs after thawing. This result confirmed the previous findings that oviduct epithelial cells-derived EVs possessed abundant transcripts of cell adhesion molecules (Alminana *et al.* 2017, Qamar *et al.* 2020).

The TE expressed several structural and functional constituents of TJ that facilitate the actively re-expanding blastocoel (Strumpf *et al.* 2005, Moriwaki *et al.* 2007). We observed the cryo-protective effect of SOF + EVs supplementation on paracellular sealing by the increase in the mRNA level of TJ assembly genes (*CLDN2, CLDN4, OCLN,*and* Actinγ2*) in cryo-thawed BLs that facilitates the BLs re-expansion. To further explore the maintenance of TE epithelial differentiation and integrity, we examined the membrane permeability function of TJ complexes and the accumulation of fluid in the re-expanded BLs. Our results revealed that the decreased mRNA level of cell junctional genes causes the insufficient function of junctional components that leads to defective paracellular sealing and results in cavity collapse in SOF cultured BLs. It was reported that TJ contacts IVP bovine embryos are more susceptible to the intense process of cryopreservation (Miller *et al.* 2003, Lonergan 2007) whereas the supplementation of EVs in the culture media restored the functional junction by influencing the transcript of TJ genes in cryo-survived embryos.

The establishment of an ionic gradient across the TE that promotes the osmotic movement of H_2_O into the formation of the blastocoel cavity is crucial for the embryo to undergo rehydration. The water channel proteins AQPs and Na^+^/K^+^ ATP1α1 have an important role in mediating the osmoregulation and fluid transport during blastocoel formation (Camargo *et al.* 2011, Somafi *et al.* 2011). There are different isoforms of mammalian AQPs (1, 2, 4, 5, 6, 8, and 10) which have permeability for water molecules while others called aqua-glyceroporins (AQPs, 3, 7, and 9) have a selectivity for both water and small solutes. During the formation of murine BL, the localization of AQP3 on the TE epithelium and its higher expression indicated a major passageway for the movement of H_2_O molecules (Barcroft *et al.* 2003). Therefore, we evaluated the re-expansion and hatching ability of surviving embryos and their relation with transcript abundance of AQP3, AQP8, AQP9, and ATP1α1. The increased expression of AQPs and reduced ATP1α1 in the EVs supplemented group makes the TE epithelium more permeable for water molecules resulting in a formation of an ionic gradient across the TE membrane that drives the H_2_O into the re-expanding blastocoel cavity. Previously it was reported that oviductal fluid containing EVs is enriched in membrane trafficking protein aquaporins that facilitate the rapid movement of H_2_O across the TE membrane after thawing of vitrified bovine embryos (Lopera-Vasquez *et al.* 2017). Thus, the presence of a relatively higher amount of AQP3 transcripts in EVs supplemented group might restore the AQP3 transcript level after freeze-thawing and also provides the reason why BLs demonstrated the different ability to undergo re-expansion and hatching when cultured in SOF and SOF + EVs medium.

**Figure 6:**
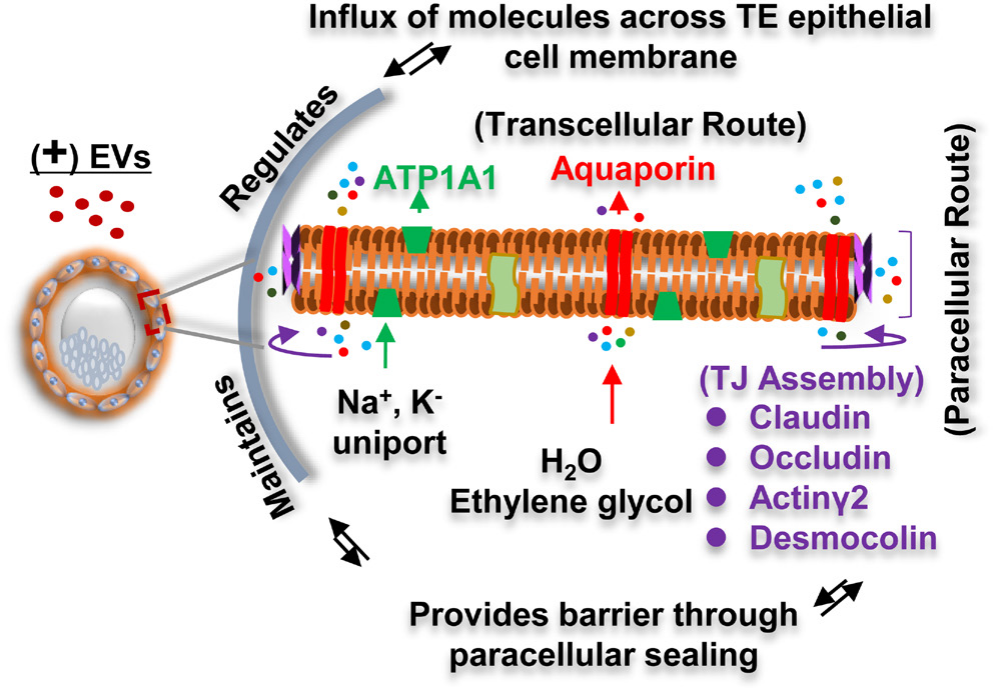
Proposed model for the effects of EVs on TJ assembly and water and ion transport for embryo blastocoel re-expansion after cryopreservation. The addition of EVs in the IVC media improves the hydraulic oscillation across the TE epithelium of the re-expanding BL. The model illustrates that the presence of EVs in the culture media strengthens the para-cellular sealing by inducing the expression of several TJ genes that maintains the accumulation of fluid influx during cavity re-expansion. The EVs supplemented BLs shows higher expression of H_2_O channel regulated proteins AQPs and vice versa reduced ATP1α1 (transport of Na^+^, K^−^-regulated ATPases) expression which promotes the inward H_2_O movement and results in active re-expansion of BLs after freeze‒thawing. Red (water) and green (ions) arrows indicate the influx of molecules across the TE membrane.

Together, our results revealed that the EVs supplemented group caused the majority of BLs to re-expand and hatch faster, whereas, in the control group, many BLs delayed the cavity re-expansion or either re-expanded but failed to hatch. Lastly, our findings are illustrated in the schematic model (Fig. 6) illustrating that the EVs supplementation in the media maintained the H_2_O and ionic osmotic flux by regulating the expression of AQPs and ATP1α1 and leads to the fluid accumulation during re-expansion of BL cavity. The absence of EVs leads to the perturbed fluid flux that impairs the BL expansion and hatching rate during the freeze‒thaw cycle. In conclusion, our findings suggest that *in vitro* cultured BOECs-derived EVs are enriched in abundance of transcripts which improves the cryo-tolerance of IVP bovine embryos by protecting the paracellular sealing and balancing the transcellular movement of H_2_O and ions across the TE epithelium which drives the re-expansion of freeze‒thawed BLs.

## Supplementary Material

Supplementary Materials

## Declaration of interest

The authors declare that there is no conflict of interest that could be perceived as prejudicing the impartiality of the research reported.

## Funding

This work was partly supported by the National Research Foundation http://dx.doi.org/10.13039/501100001321 of Korea (NRF), grant funded by the Korean government (MSIT no. 2020R1A2C2006614), Korean Institute of Planning, by the Technology and Information Promotion Agency for SMEs (IPA), grant funded by the Korean government (S2971179), and by the R&D Innovation Cluster, grant funded by the Korean government Innovation Foundation (grant no: 2020-IT-RD-0193-01-101), Republic of Korea.

## Authors contribution statement

T S, conceptualization, methodology, and data curation. A A K and T S writing of the original draft. A A K writing, revision, and editing of the draft. M-D J management and collection of bovine ovaries. L X helped with the statistical analysis. M E S and J-H K helped with reagent preparations. I-K K funds acquisition and supervised the whole project. All authors have read and agreed to the published version of the manuscript.
