# Supplementary material for: Extracellular vesicles improve embryo cryotolerance by maintaining the tight junction integrity during blastocoel re-expansion
Source: Reproduction. 2022 Feb 7;163(4):219–32. doi: 10.1530/REP-21-0320 (PMC8942337; doi:10.1530/REP-21-0320)
Supplement: Supplementary Materials [file supplementary_material.pdf]

Title

Extracellular vesicles improve embryo cryotolerance by maintaining the tight junctional integrity during blastocoel re-expansion.

Tabinda Sidrat[1+], Abdul Aziz Khan[2+], Myeong-Don Joo[1], Lianguang Xu[1], Marwa El Sheikh[1,3], Jong-Hyuk Ko[4], and Il-Keun Kong[1,4,5*]

Supplementary Table S1: List of qRT-PCR primers.

Gene Name	Forward	Reverse	Accession #
GAPDH	TTCAACGGCACAGTCAAGG	ACATACTCAGCACCAGCATCAC	NM_001034034
PlAC8	TGAGGACCCTCTACAGGACTCG	TAGAAAGTGCGATTGGCTCTCC	XM_024993443.1
IGF-1R	GATCCCGTGTTCTTCTACGTTC	CAGCCTGCTGCTATTTCTTTTT	NM_001244612.1
OCT4	AGGTGTTCAGCCAAACGACTAT	GTCTCTGCCTTGCATATCTCCT	NM_174580
INF-Ʈau	CCCCATTCTGACTGTGAAGA	TTTTGCAAGGTGGTTGATGA	AF238612.1
MnSOD2	CGCTGGAGAAGGGTGATGTTAC	GTTTGATGGCTTCCAGCAATTC	NM_201527.2
GPX4	GCACGAATTTTCAGCCAAGG	AAACCACACTCGGCGTATCG	NM_001346431.1
HSP70	CAACGTGCTCATCTTTGATCTG	GTCCTTCTTGTGCTTCCTCTTG	NM_203322.3
HSP90	TGTCTGACGATGAAGACGAGAC	CTCCTTCTCTTTCTGGAACTGC	NM_001035338.2
CD44	CCGGAACATAGGGTTTGAGA	GGTATAACGGGTGCCATCAC	NM_174013.3
CDH1	CCGTGAGAGTTTTCCCACAT	CATTGGTGACTGGGTCTGTG	AY508164.1
CLDN2	CTTTACCTGGGCATTATTTCCTC	AGTTAAACTCACTCTTGCCTTTCG	NM_205781.2
CLDN4	GTCATCCGCGACTTCTACAAC	AGTACTTAGCGGAGTAGGGCTTG	NM_001014391.2
OCLN	AATCACTACACACCAAGCAATGAC	AAGCATAGACAGGATCCGAATTAC	NM_001082433.2
ACTINγ2	AAGAGCTACGAGCTGCCAGATG	GCGGATGTCAATGTCACACTTC	BT021005.1
AQP3	GTACCTCAATGGGCTTCAACTC	ATCATGAGCTGGTACACGAAGAC	NM_001079794.1
AQP8	CACTGGGACTACCACTGGATCTAC	TCACCGTCCCTTTAGGATTAGAC	NM_001206607.3
AQP9	AACTTCTGGTGGATTCCTGTAGTG	ATATTTCTCTGGTTTGTCCTCTGG	NM_001205833.3
ATP1α1	TATGGACAGATTGGTATGATCCAG	ATCTTCCTCTGTTCATAGGTCCAC	NM_001076798.1

Supplementary Table S2: List of Antibodies used.

Target	Cat #	Manufacturer
Anti-CDX2	B-8434	Sigma
Anti-CD44	ab157107	Abcam
Anti-AQP3	ab153694	Abcam
Anti-ATP1α1	MA3-928	Invitrogen
DAPI	62248	Thermo Fisher scientific
FITC	B2119	Santa Cruz
TRITC	A6071	Invitrogen
